# Reduced cardiovascular and metabolic responses during eccentric stepping exercise: A pilot study

**DOI:** 10.14814/phy2.70080

**Published:** 2024-10-06

**Authors:** Nicholas C. Renwick, Stuart Egginton, Carrie Ferguson

**Affiliations:** ^1^ School of Biomedical Sciences, Faculty of Biological Sciences University of Leeds Leeds UK; ^2^ The Lundquist Institute for Biomedical Innovation at Harbor‐UCLA Medical Center Torrance California USA

**Keywords:** cardiorespiratory, concentric exercise, eccentric exercise, negative work, rehabilitation

## Abstract

This study compared cardiovascular and metabolic responses during concentric and eccentric stepping. Eight participants (5 m, 3f; 22 ± 2 years) performed maximal concentric and eccentric ramp incremental tests on a modified stepping ergometer. Subsequently, three randomized 15‐min constant‐power tests were performed (1) concentric stepping at 90% of the concentric lactate threshold (LT), (2) eccentric stepping at the same power, and (3) eccentric stepping at the same oxygen uptake (V̇O_2_). At equivalent power (36 ± 6 W, *p* = 0.62), eccentric stepping resulted in 46 ± 8% lower V̇O_2_, 16 ± 6% lower heart rate (HR), and 11 ± 5% lower mean arterial blood pressure compared to concentric (*p* < 0.01). Matching V̇O_2_ required 65 ± 19% more power during eccentric stepping (*p* < 0.01). During this test, eccentric V̇O_2_ and HR continued to increase, resulting in a 22 ± 29% higher V̇O_2_ and 19 ± 16% higher HR in the final minute (*p* < 0.001). Reduced cardiorespiratory demand during eccentric stepping at the same power as concentric demonstrates a higher eccentric power is required to produce the same V̇O_2_. However, despite being below the concentric LT, eccentric V̇O_2_ and HR continued to increase past the predicted steady state, indicating a higher exercise intensity.

## 1 INTRODUCTION

Eccentric exercise (muscle lengthening during contraction) is an effective training modality both in athletes to improve sporting performance, and exercise intolerant populations to provide functional improvements related to quality of life (QoL) (Isner‐Horobeti et al., 2013; Križaj et al., 2022). Compared with concentric exercise (muscle shortening during contraction), eccentric exercise performed at similar or lower metabolic requirement induces greater increases in muscle strength and cross‐sectional area (Isner‐Horobeti et al., 2013; Roig et al., 2009), providing greater improvements in athlete specific (Gross et al., 2010), and rehabilitation related (LaStayo et al., 2003) outcomes. Greater strength increases with eccentric exercise are primarily due to the ability to resist greater mechanical loads at all contraction velocities (Hill, 1938; Westing et al., 1990). Additionally, studies report an increase in muscle fiber cross‐sectional area and increases in muscle strength, with one study reporting a 36% increase in leg strength and a 52% increase in cross‐sectional area (LaStayo et al., 2000). Importantly, these benefits are associated with an acute reduction in cardiovascular (heart rate) and metabolic (oxygen uptake, V̇O_2_) responses compared to concentric exercise performed at the same external mechanical power. Specifically, heart rate may be up to 35% lower and V̇O_2_ up to 70% lower (Isner‐Horobeti et al., 2013).

The majority of exercise rehabilitation advocated for patients with reduced exercise capacity (e.g., older adults, chronic obstructive pulmonary disease (COPD), and chronic heart failure (CHF) patients) consists of concentric or mixed forms of exercise (Ries et al., 2007). However, adherence to rehabilitation programs are poor (Hayton et al., 2013), potentially due to (1) the high pulmonary and cardiovascular requirements to meet the metabolic demands of these protocols leading to a high symptomatic burden, and (2) the slow progression of noticeable strength gains and mobility improvements. Therefore, eccentric exercise may provide a modality that results in greater strength and functional mobility improvements but with less symptomatic burden during the exercise, leading to increased adherence, mobility and ultimately QoL.

Eccentric exercise research has primarily used reverse cycle ergometry (Isner‐Horobeti et al., 2013; Križaj et al., 2022), which involves the participant resisting the reverse movement of motor driven bike cranks. This modality enables tight control of exercise parameters, but lacks ecological validity due to large differences between natural locomotion and reverse pedaling. A custom eccentric stepping ergometer may provide an exercise modality that more closely mirrors downhill walking or stair descent whilst being able to maintain tight control of exercise parameters (Tinwala et al., 2017). Therefore, this modality may provide a more functional form of exercise that translates into greater improvements in activity specific strength and mobility (Misic et al., 2009; Morrissey et al., 1995). However, the effect of eccentric versus concentric stepping on the HR and V̇O_2_ responses are unknown. It is also unknown whether eccentric stepping performed at the same concentric V̇O_2_ can be tolerated to allow a greater external mechanical power to be sustained throughout a training session.

The purpose of this study was to compare concentric and eccentric responses during stepping exercise performed at: (1) the same external mechanical power, (2) the same V̇O_2_. We hypothesized that: (1) cardiovascular and metabolic responses would be reduced during eccentric stepping performed at the same power as concentric stepping, and (2) eccentric stepping at the same concentric V̇O_2_ would be tolerable and allow a greater power to be sustained during an exercise session.

## 2 METHODS

### 2.1 Participants

Eight healthy participants (5 male, 3 female; mean ± SD; 22 ± 2 years, 70 ± 9 kg, 172 ± 6 cm) were recruited from the University of Leeds via emails, posters, and personal contact. All volunteers provided written informed consent and were screened with a health and physical activity questionnaire. Individuals identified as having any known disease that may affect physiologic responses, or any contraindications for exercise were excluded. Post hoc power analysis determined that our study has a power of 1.0 to detect a difference in V̇O_2_ between conditions at a significance level of 0.05, principally due to the large effect size obtained (partial eta squared value of 0.904; G*Power 3.1.9.2; www.gpower.hhu.de).

### 2.2 Eccentric ergometer

All exercise testing was performed on a recumbent stepping ergometer (Eccentron; BTE, Hanover, MD, USA) modified to allow both concentric and eccentric muscle contractions without changing any parameters such as seat position, joint angles, stride length (degree of displacement between foot plates), or cadence (Renwick, 2017). Raw left and right force (N) data were measured directly from calibrated load cells behind the foot plates and were fed into a data acquisition system (ML870 PowerLab 8/30; ADInstruments Ltd., Oxford, UK). Real‐time assessment and quantification of force and power were performed using programmable software (LabChart 8; ADInstruments Ltd., Oxford, UK). To reduce the possibility of knee hyperextension during loading, the seat position was set so that maximal knee extension was 30°. Cadence was set at 23 steps per minute, a rate found to be optimal for participant control of force development. Ergometer settings remained the same for each participant throughout testing. Participants viewed a custom LabChart screen displaying left and right cumulative work performed (area under the force curve), superimposed against a target band that identified ±2% of the target cumulative work.

### 2.3 Familiarization

Participants attended familiarization sessions before the commencement of formal testing. This was conducted to ensure participants were executing the contractions correctly and could accurately maintain the target power, as well as to reduce the severity of delayed onset muscle soreness (DOMS), and anxiety responses. Familiarization was identical to the ramp incremental test (RIT) protocol; however, participants were instructed to only continue up to 80% of their self‐perceived maximum. Participants performed both concentric and eccentric RITs. Participants were deemed familiarized when they were able to maintain cumulative work within ±2% of the target for more than 2 min.

### 2.4 Exercise testing

Following familiarization, participants visited the laboratory on five separate occasions (two RITs followed by three constant‐power tests) with a minimum separation of 48 h between tests. Before each visit, participants abstained from strenuous exercise (previous 24 h), alcohol consumption (24 h), food, and caffeine ingestion (3 h).

#### 2.4.1 Ramp‐incremental tests

Participants initially undertook a concentric and eccentric RIT to the limit of tolerance in a randomized order. A 3‐min 20 W warm‐up preceded a linear increase in power of 7 W min^−1^. The limit of tolerance was identified as the point at which, despite strong verbal encouragement, left and/or right cumulative work remained below the target band (± 2%) for five consecutive contractions. At the limit of tolerance, the target power was reduced to 20 W for a 5‐min recovery period.

#### 2.4.2 Constant‐power tests

**FIGURE 1:**
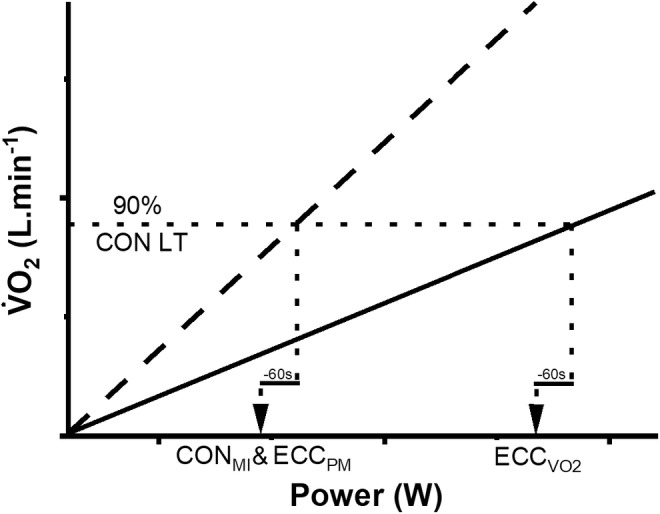
Protocol for determining target power for the constant‐power tests. The intercept of the target V̇O_2_ (dotted line) with the concentric RIT V̇O_2_ response (dashed line) provided the power required for the concentric moderate intensity (CON_MI_), and eccentric power‐matched (ECC_PM_) conditions. The intercept with eccentric ramp‐incremental V̇O_2_ response (solid line) provided the power required for the eccentric V̇O_2_‐matched condition (${\mathrm{ECC}}_{\dot{V}O_{2}}$). Sixty seconds of power was subsequently subtracted to allow for the MRT of the V̇O_2_ response (horizontal solid line).

Three constant‐power tests were then performed in a randomized order to match for both power and V̇O_2_ between concentric and eccentric stepping exercise (Figure 1). To determine required power, the lactate threshold (LT) of the concentric RIT was measured using non‐invasive gas‐exchange criteria (Beaver et al., 1986). The power required to achieve 90% of the concentric LT (moderate‐intensity exercise) was then determined using the V̇O_2_/power relationship of the concentric RIT. Sixty seconds of work (i.e., 7 W, based on the 7 W min ramp‐rate) was subtracted to allow for the mean response time of V̇O_2_ kinetics and obtain the correct power to achieve the target steady‐state V̇O_2_ (Whipp et al., 1981). This moderate‐intensity power was then used during concentric (CON_MI_) and power‐matched eccentric (ECC_PM_) constant‐power tests. The eccentric power required to attain the same V̇O_2_ (90% of concentric LT) was then determined from the V̇O_2_/power relationship of the eccentric RIT and used for the third V̇O_2_‐matched constant‐power test (${\mathrm{ECC}}_{\dot{V}O_{2}}$). A 3‐min 20 W warm‐up preceded a step increase in target power for 15 min. This was followed by a 5‐min recovery period at a target power of 20 W.

### 2.5 Measurements

A breath‐by‐breath pulmonary gas analysis system (MedGraphics D‐Series; Medical Graphics Corporation, St Paul, MN, USA) was used to measure expired flow and gas concentrations to and provide a measure of ventilatory and pulmonary gas exchange responses throughout all tests. Before each test, the flow sensor was calibrated using a syringe (Medical Graphics Corporation) to inject 10 × 3.0 L volumes at varied flow rates encompassing normative ventilation ranges between rest and peak exercise. A mean volume within the range of 2.98–3.02 L was used as the acceptance criteria. Gas analyzers were calibrated using certified known gas concentrations (CO_2_ 0% and 5%, O_2_ 12% and 21%; Medical Graphics Corporation) and re‐sampled immediately post‐test to confirm that no excess drift occurred. Participants breathed through a mouthpiece and wore a nose clip throughout testing to ensure all expired gas was captured and was connected to the gas analysis system via an umbilical sample line.

Raw left and right force (Newtons (N)) data were obtained at a rate of 1 KHz from calibrated load cells located directly behind the foot plates. Data are presented in Watts (W) to replicate previous eccentric research. Power comparison between stepping and cycling is not possible due to vastly different ergometer mechanics. As cadence and stride length were kept constant throughout testing, the angular velocity of the foot plates could be calculated, and power determined using the relationship:

$$\text{Power}\;\left(W\right)=\text{Force}\;\left(N\right)\times 0.094$$

Electrocardiogram (ECG) data were obtained using a 3‐lead ECG bioamplifier unit (FE132 Bio Amp, ADInstruments Ltd., Oxford, UK), visualized and analyzed on LabChart software, and the R‐R interval used to determine HR throughout all protocols.

Systolic blood pressure (SBP) and diastolic blood pressure (DBP) were obtained every 30 s throughout testing using an automatic digital brachial cuff device (UA‐787, A&D Medical, Abingdon, UK). The participant's arm was positioned to the side of the body with the cuff placed at heart level. Mean arterial blood pressure (MAP) was calculated as DBP + 1/3(SBP‐DBP).

### 2.6 Data analysis

Breath‐by‐breath V̇O_2_ data were initially filtered by removing data points > ± 3 SD about the mean (13). This aimed to exclude any erroneous breaths caused by swallowing, coughing or sighing which lay outside of the expected underlying physiologic responses. Peak V̇O_2_ was identified as the maximum 12‐breath moving average from the final 20 breaths of the RIT. Breath‐by‐breath data were subsequently linearly interpolated to give a value every second and allow the calculation of the 1‐min mean. To determine gain of the V̇O_2_ (∆V̇O_2_/∆W) and HR (∆HR/∆W) RIT responses, linear regressions were fit to the linear phase of the RIT response. For the constant‐power tests, differences between the 3rd (sufficient time to allow a steady state to be attained during moderate‐intensity exercise in healthy participants (14)) and 15th minute means were calculated to quantify any further increase in V̇O_2_ and HR during the exercise.

### 2.7 Statistical analysis

Results are expressed as means ± SD. All statistical analyses were completed using GraphPad Prism (GraphPad v6.0, GraphPad Software, Inc., CA, USA) with differences significant when *p* < 0.05. All data passed normality testing using the D'Agostino & Pearson omnibus normality test. Paired Students *t*‐tests were used to compare concentric and eccentric RIT responses. For the constant‐power tests, two‐way repeated measures analysis of variance (ANOVA) was conducted to compare the main effects of time and exercise condition on the V̇O_2_ and HR responses, whilst a one‐way ANOVA was used to compare the 10‐min mean MAP response between exercise conditions. To reduce the risk of type I error, Bonferroni corrections were used for all post‐hoc comparisons.

## 3 RESULTS

### 3.1 Ramp incremental tests

Participants required between one and three familiarization sessions to be able to maintain cumulative work within ±2% of the target for ≥2 min, and progress to the formal RITs. Comparison of concentric and eccentric responses during the RITs are summarized in Table 1. The rate of increase (gain) in HR (∆HR/∆W) and V̇O_2_ (∆V̇O_2_/∆W) was 40 ± 11% and 49 ± 14% lower during eccentric, compared with concentric RIT exercise, respectively (*p* < 0.001). The mean concentric LT was 1.28 ± 0.14 L.min^−1^, 58 ± 3% of the concentric peak V̇O_2_. Peak power achieved at the RIT limit of tolerance was higher during eccentric stepping (*p* = 0.01). There was a lower eccentric absolute and relative peak V̇O_2_, compared with that attained in the concentric test (*p* < 0.001), however there was no difference in peak HR achieved between contraction types.

**TABLE 1:** Concentric (CON) and eccentric (ECC) cardiovascular and metabolic responses to ramp incremental stepping exercise.

	CON	ECC	ECC (% of CON)
Peak V̇O_2_ (L.min^−1^)	2.21 ± 0.25	1.75 ± 0.30*	79 ± 9
Peak V̇O_2_ (mL.min^−1^. kg^−1^)	32.0 ± 4.4	25.1 ± 4.4*	79 ± 9
Peak HR (beats.min^−1^)	159 ± 14	150 ± 20	94 ± 9
Peak power (W)	68 ± 12	87 ± 23*	128 ± 17
V̇O_2_ gain (mL.min^−1^.W^−1^)	68 ± 13	34 ± 9*	51 ± 13
HR gain (beats.min^−1^.W^−1^)	3.3 ± 0.9	2.0 ± 0.7	60 ± 11

*Note*: Results are expressed as means ± SD, *n* = 8.

* Significantly different (*p* < 0.05) versus concentric exercise.

### 3.2 Constant‐power tests

**FIGURE 2:**
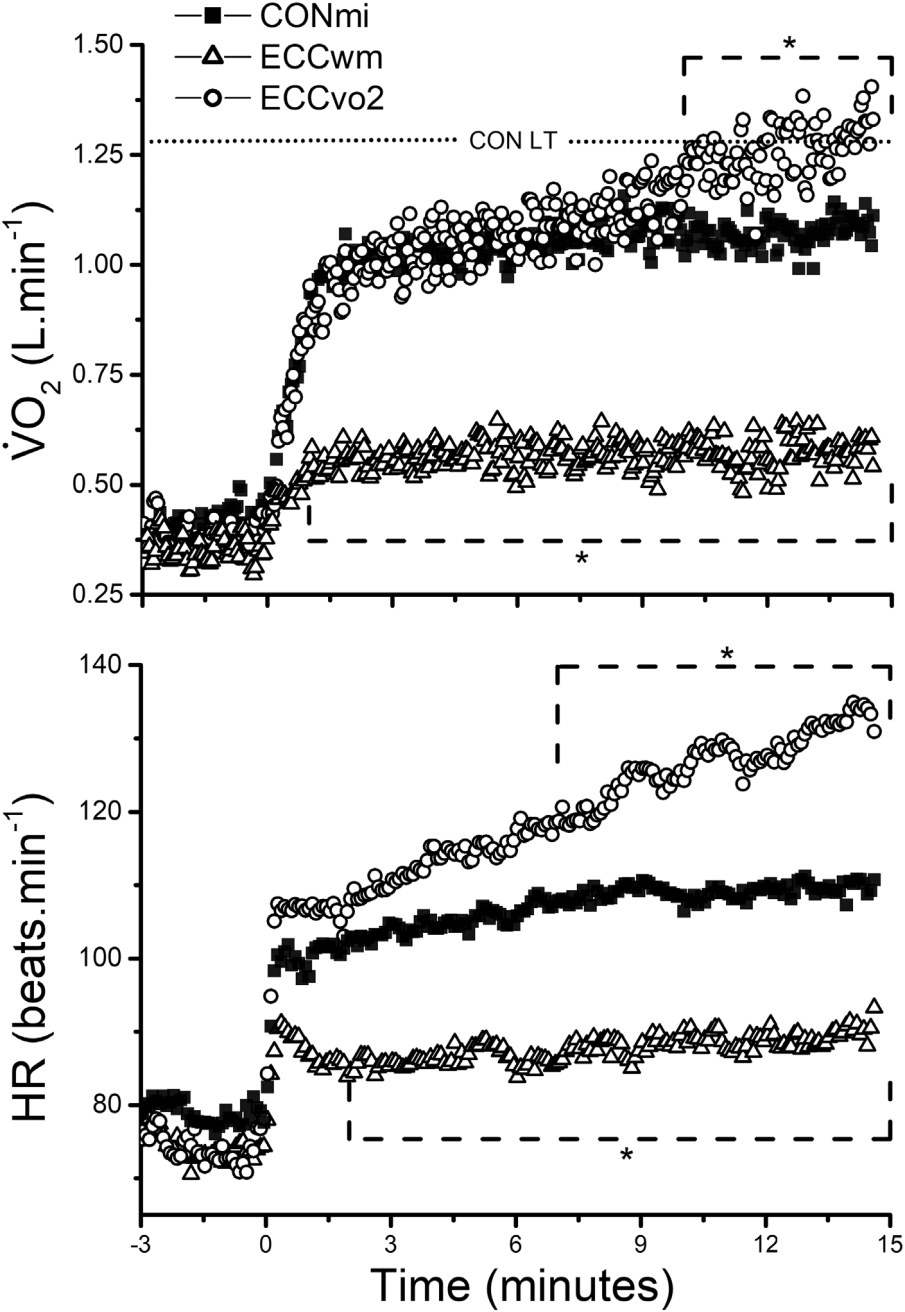
Mean interpolated oxygen uptake (V̇O_2_) and heart rate (HR) (5 s rolling mean) during the 15‐min constant‐power tests. Dotted line indicates the mean concentric lactate threshold (LT). * Significantly different (*p* < 0.05) versus CON_MI_ for each minute within the dashed line region. *n* = 8.

There was less than a 0.4% difference between the target and actual power performed during all tests (0.28 ± 0.16% (CON_MI_), 0.39 ± 0.12% (ECC_WM_), 0.34 ± 0.19% (${\mathrm{ECC}}_{\dot{V}O_{2}}$); all n. s.). At the same mechanical power (36 ± 6 (CON_MI_) versus 36 ± 6 (ECC_PM_) W; *p* = 0.62), both concentric and eccentric V̇O_2_ and HR attained a steady state after 3 min (Figure 2). Comparing the responses in the final minute, ECC_PM_ V̇O_2_ was 0.52 ± 0.17 L.min^−1^ lower (46 ± 8%), and HR was 19 ± 9 bpm lower (16 ± 6%) compared with CON_MI_ (*p* < 0.0001 and *p* < 0.01, respectively; Figure 2; Table 2). MAP was 11 ± 5 mmHg lower (11 ± 5%) during ECC_PM_ compared with concentric stepping at the same power (CON_MI_; 103 ± 6 versus 92 ± 6 mmHg; *p* < 0.01).

**TABLE 2:** Final minute mean oxygen uptake (V̇O_2_), heart rate (HR), ventilation (V̇E) and respiratory exchange ratio (RER) during the constant power tests [concentric moderate intensity (CON_MI_), eccentric power‐matched (ECC_PM_), eccentric V̇O_2_‐matched (${\mathrm{ECC}}_{\dot{V}O_{2}}$)].

	CON_MI_	ECC_PM_	${\mathrm{ECC}}_{\dot{V}O_{2}}$
V̇O_2_ (L.min^−1^)	1.10 ± 0.20	0.58 ± 0.12*	1.34 ± 0.17*
HR (beats.min^−1^)	112 ± 13	93 ± 8*	133 ± 18*
V̇E (L.min^−1^)	28.8 ± 5.5	16.7 ± 2.5*	36.9 ± 7.5*
RER	0.89 ± 0.06	0.83 ± 0.06	0.86 ± 0.06

*Note*: Results are expressed as means ± SD, *n* = 8.

* Significantly different (*p* < 0.01) versus CON_MI_.

To match the eccentric V̇O_2_ to that achieved during concentric stepping, eccentric power had to be increased by 65 ± 19% (60 ± 13 W; *p* < 0.001) and was tolerated by all subjects for the full 15‐min duration. Despite this increase in power, MAP was not different compared with CON_MI_ (103 ± 6 (CON_MI_) vs. 95 ± 15 (${\mathrm{ECC}}_{\dot{V}O_{2}}$) mmHg; *p* = 0.26). When comparing 1‐min mean intervals, concentric and eccentric V̇O_2_ and HR were initially matched (Figure 2), however, the ${\mathrm{ECC}}_{\dot{V}O_{2}}$ test did not reach steady state. Instead, V̇O_2_ continued to increase throughout the 15‐min test, with a ∆V̇O_2_ of 0.33 ± 0.15 L.min^−1^ between 3 and 15 min (*p* < 0.001). This resulted in the eccentric V̇O_2_ and HR exceeding that of the concentric test from 10 and 7 min, respectively (both *p* < 0.05; Figure 2). This led to a 0.23 ± 0.3 L.min^−1^ higher eccentric V̇O_2_ and a 21 ± 17 bpm higher eccentric HR versus CON_MI_ in the final minute (both *p* < 0.001; Table 2).

Within the final minute, there was a lower eccentric O_2_ pulse (V̇O_2_/HR) when matched for mechanical power (ECC_PM_ vs. CON_MI_; *p* < 0.01). The O_2_ pulse was not different when matching for metabolic requirement (${\mathrm{ECC}}_{\dot{V}O_{2}}$ vs. CON_MI_).

## 4 DISCUSSION

This study compared concentric and eccentric cardiovascular and metabolic responses during novel stepping exercise performed at (1) the same external mechanical power and (2) the same predicted V̇O_2_. For the first time, we demonstrated that during eccentric stepping (1) the RIT V̇O_2_ and HR gain is reduced compared with concentric stepping; (2) V̇O_2_, HR, and MAP are lower during eccentric versus concentric stepping at the same external mechanical power (ECC_PM_ vs. CON_MI_); (3) to match eccentric V̇O_2_ to the response seen during concentric constant power stepping, external mechanical power had to be increased by 65% (i.e., ${\mathrm{ECC}}_{\dot{V}O_{2}}$ vs. CON_MI_); (4) despite initial matching of V̇O_2_ (CON_MI_ vs. ${\mathrm{ECC}}_{\dot{V}O_{2}}$), there was a delayed increase within the ${\mathrm{ECC}}_{\dot{V}O_{2}}$ test, suggesting this test was of a higher exercise intensity.

Our data demonstrates that a novel eccentric stepping ergometer, which may provide a more ecologically valid form of eccentric exercise than previous modalities, is appropriate for use within a research setting. All subjects became familiarized to this modality within three sessions, a comparable or faster time compared with other eccentric modalities (Beaven et al., 2014; Dufour et al., 2004), thus demonstrating participants can routinely achieve the desired power in a controlled manner throughout the tests. The ergometer allowed for similar joint angles and stride lengths to that of stair ascent and decline (Costigan et al., 2002; McFadyen & Winter, 1988; Samuel et al., 2011), likely to result in greater translation to increases in muscle strength and functional performance than reverse cycle ergometry (Misic et al., 2009; Morrissey et al., 1995). This modality also provides greater safety precautions than other available eccentric ergometers, such as greater control of knee joint angles, emergency stop buttons, and pads underneath the knees that serve to prevent hyperextension and potential damage to the knee joint.

To our knowledge, this is the first study to utilize maximal RIT stepping exercise (analogous to standard RIT cycle ergometry tests). The reduction in eccentric V̇O_2_ and HR gain (∆V̇O_2_/∆WR, ∆HR/∆WR) observed during RIT stepping exercise are consistent with previous reverse cycle ergometry studies, however, the magnitude of these reductions was less than predicted. For example, one study demonstrated that eccentric V̇O_2_ and HR gain (ml.min^−1^.W^−1^ and beats.min^−1^.W^−1^) were just ~25% and ~ 45% of the concentric gain responses (data extrapolated from (Dufour et al., 2004)). This is in contrast to our 51 ± 13% and 60 ± 11% of concentric responses, respectively. Regardless, the lower eccentric V̇O_2_ and HR gain allowed participants to achieve a greater eccentric power for a given V̇O_2_ and HR throughout the test, and a higher peak power (~28% greater) at the limit of tolerance compared to concentric exercise. Despite this increase in power, eccentric peak V̇O_2_ at the limit of tolerance was 21 ± 9% lower than the concentric RIT. As the eccentric RIT test did not encroach on the pulmonary and cardiovascular limits established within the concentric RIT, we hypothesize that the eccentric limit of tolerance was likely determined by a peripheral inability to generate the higher target power, rather than a limitation in the ability to deliver and/or utilize O_2_.

Despite the higher peak power, and lower peak V̇O_2_ during eccentric stepping, peak HR was not different between concentric and eccentric tests. This meant at a given V̇O_2_ during eccentric exercise, HR was greater, with the reason for this unclear, but evidence suggests that the higher forces required to match V̇O_2_ likely increase muscle tension and temperature evoking a greater increase in muscle afferent feedback (Thomson, 1971). The constriction of muscle and stretch of associated tendons has also been shown to increase type III and IV muscle afferent firing rates, subsequently triggering an increase in HR via feedback to the nucleus tractus solitarii of the medulla oblongata (Craig, 1995; Kaufman et al., 1983). Eccentric exercise is known to result in increases in muscle temperature (Sargeant & Dolan, 1987) and cat models have shown that an elevated temperature increases firing rate of both type III and IV muscle afferents (Hertel et al., 1976), likely to stimulate similar increases in HR.

During constant power tests, when matching for external mechanical power, there was a ~ 46 ± 8% lower V̇O_2_, a ~ 16 ± 6% lower HR and an ~11 ± 5% lower MAP during eccentric compared to concentric stepping. This reduction in cardiovascular and metabolic responses during eccentric stepping, is consistent with data from studies using reverse cycle ergometry (Dufour et al., 2004; LaStayo et al., 1999; Perrey et al., 2001). Using the LT to set target power ensured the concentric test was of a moderate‐intensity (Whipp & Wasserman, 1972). However, this exercise intensity makes comparing the magnitude of reductions in cardiovascular and metabolic responses with those of other studies difficult, as some of these tests were performed at higher intensities (e.g., ~3.8 L.min^−1^ (Dufour et al., 2004)), that affect the gain of the V̇O_2_/power relationship (Ozyener et al., 2001).

The reduction in V̇O_2_ gain during the eccentric RITs meant that in order to attain the same V̇O_2_ as the CON_MI_ test, the external mechanical power had to be increased by ~65%. This is a smaller increase in power than has been reported during eccentric cycle ergometry, with increases of between 412% (Hesser et al., 1977) and 500% (Dufour et al., 2004) used previously to attain the required V̇O_2_. This large difference is likely a result of variances in ergometer mechanics, and/or the lower cadences utilized within this study. It has previously been shown that, by increasing cadence from 30 to 100 rpm, the concentric: eccentric V̇O_2_ gain changes from 5:1 to 10:1 (Bigland‐Ritchie & Woods, 1976), and with normal cadences within reverse cycle ergometry being over twice that used within this study (23 vs. ~60 rpm; LaStayo et al., 2000), this may partly provide a possible explanation for these results.

Although V̇O_2_ and HR were initially matched between CON_MI_ and ${\mathrm{ECC}}_{\dot{V}O_{2}}$ conditions, eccentric V̇O_2_ and HR continued to rise throughout the 15 min and failed to reach a steady state. This suggests that, despite matching for 90% of CON LT (moderate‐intensity domain), the ${\mathrm{ECC}}_{\dot{V}O_{2}}$ tests were performed at a higher exercise intensity (Rossiter, 2011). This delayed increase may imply the addition of a V̇O_2_ slow component during the eccentric test (Whipp & Wasserman, 1972). This represents a progressive reduction in work efficiency, causing a delayed steady state when exercising within in the heavy domain, and attainment of V̇O_2_ max when exercising above critical power (the intensity demarcator above which lactate and V̇O_2_ cannot achieve steady state) (Whipp, 1994). As a steady state was not achieved within the test, it remains unclear whether this eccentric stepping exercise was performed within the heavy or very heavy intensity domains, indicating the importance of measuring blood lactate concentration during eccentric stepping in future experiments.

Compared with concentric stepping, MAP was lower during eccentric stepping at the same power (ECC_PM_). Interestingly, MAP was reduced to a similar extent when the eccentric power was increased to match the V̇O_2_ of the concentric test (${\mathrm{ECC}}_{\dot{V}O_{2}}$). This reduction in BP during eccentric stepping is consistent with previous eccentric cycling studies (Overend et al., 2000; Vallejo et al., 2006), and hence may provide a safer exercise modality than traditional (concentric) exercise for some clinical populations. A lower eccentric BP response may be the result of the exercise pressor reflex being more closely linked to perceived effort and central command than force production and subsequent muscle afferent feedback (MacDougall et al., 1992).

The reduced eccentric cardiovascular and metabolic responses both within the RITs and constant‐power tests suggests a reduction in the energy required to perform the same amount of mechanical work. The cross‐bridge theory of eccentric contractions alone cannot explain these findings, which has led to numerous alternate theories aiming to explain this reduced V̇O_2_, with the most plausible being the mechanical breaking of cross bridges, the inclusion of the partner myosin head, and addition of the protein titin to passive force contribution. First, the mechanical breaking of cross bridges during eccentric contractions is thought to eliminate the need for ATP to bind and cause dissociation of actin from myosin (Stauber, 1988). This breaking of cross bridges may provide an additional passive force contribution and leave the myosin head in an active state allowing faster reattachment to actin. The second theory suggests that during active lengthening, distortion of the myosin molecule allows additional binding of actin to the partner myosin head (Brunello et al., 2007), increasing the total number of attached cross‐bridges and, therefore, force production. Finally, it is hypothesized that in the presence of Ca^2+^ during active lengthening, the protein titin may bind to actin (Nishikawa, 2016) or myosin end filaments (DuVall et al., 2017), resulting in increased titin stiffness and increased passive force production in the absence of ATP.

This study has several limitations that should be acknowledged. First, although the study achieved a power of 1.0 to detect a difference in V̇O_2_ between conditions, the small sample size (*n*) and narrow age range of the participants make it difficult to generalize these findings to the broader population, particularly clinical populations. To confirm our findings and increase the external validity of the results, further studies with a larger and more diverse group of participants are necessary. Second, differences in ergometer mechanics make power comparisons between eccentric stepping and reverse cycle ergometry problematic. Finally, measures of perceived exertion during eccentric stepping were not obtained, preventing internal comparisons between concentric and eccentric stepping, and external comparisons to other eccentric modalities.

Based on our findings, future work should aim to assess the training stimulus provided by eccentric stepping versus other eccentric modalities, both within healthy and exercise intolerant (either from aging or pulmonary/cardiovascular/skeletal muscle limited) populations. Eccentric exercise rehabilitation is not routinely used within exercise intolerant populations that would likely benefit. This is in part due to the current lack of robust experimental evidence, as well as the cost, safety concerns, and availability of suitable eccentric training equipment (Hoppeler, 2015). The ability to produce greater mechanical power during eccentric stepping for the same concentric V̇O_2_ may allow participants with reduced pulmonary or cardiovascular function to exercise without prohibitive sensations such as dyspnoea, and benefit from improved strength, mobility and ultimately QoL.

## AUTHOR CONTRIBUTIONS

Experiments were carried out at the faculty of Biological Sciences, University of Leeds. **Nicholas C. Renwick**: Acquisition, analysis, and interpretation of data; drafting of the work and revising it critically for important intellectual content. **Nicholas C. Renwick, Carrie Ferguson, Stuart Egginton**: Conception and design of experiment, analysis, and interpretation of data; critical review of important intellectual content, revisions of research article. All authors listed above approved the final version of the manuscript and agree to be accountable for all aspects of the work in ensuring that questions related to the accuracy or integrity of any part of the work are appropriately investigated and resolved. All persons designated as authors qualify for authorship, and all those who qualify for authorship are listed.

## FUNDING INFORMATION

Funding for this study was received via the faculty of Biological Sciences, University of Leeds.

## CONFLICT OF INTEREST STATEMENT

Carrie Ferguson is supported by a grant from NIH (R01HL166850). She is involved in contracted clinical research with United Therapeutics, Genentech, Regeneron, Respira Therapeutics and Mezzion. She reports consulting fees from Respira Therapeutics. She is a visiting Associate Professor at the University of Leeds, UK.

## ETHICS STATEMENT

This study was approved by the Faculty of Biological Sciences Ethical Committee for non‐clinical research (University of Leeds, BIOSCI 13–028) and complied with the latest version of the Declaration of Helsinki, with the exception of being registered as a clinical trial.

## Data Availability

The data that support the findings of this study are available from the corresponding author upon reasonable request.
